# The Effect of Different Traditional Chinese Exercises on Blood Lipid in Middle-Aged and Elderly Individuals: A Systematic Review and Network Meta-Analysis

**DOI:** 10.3390/life11070714

**Published:** 2021-07-19

**Authors:** Yanan Gao, Lei Yu, Xiaohan Li, Chen Yang, Aiwen Wang, Huiming Huang

**Affiliations:** 1 Faculty of Sports Science, Ningbo University, Ningbo 315211, China; gaoyanan19971225@163.com (Y.G.); llisport@163.com (L.Y.); leexiaohan996@gmail.com (X.L.); wangaiwen@nbu.edu.cn (A.W.); 2 Research Academy of Grand Health, Ningbo University, Ningbo 315211, China; 3 Department of Kinesiology and Physical Education, McGill University, Montreal, QC H2W 1S4, Canada; chen.yang4@mail.mcgill.ca

**Keywords:** traditional Chinese exercises, dyslipidemia, middle-aged and aged, exercise therapy, network meta-analysis

## Abstract

Although the impact of physical exercise on blood lipids is well documented, less information is available regarding the effect of traditional Chinese exercises (TCEs), and it is unclear what the best TCE treatment for dyslipidemia in middle-aged and elderly individuals is. The aim of this study was to systematically assess the effects of TCEs (Taijiquan, TJQ; Wuqinxi, WQX; Baduanjin, BDJ; Liuzijue, LZJ; Yijinjing, YJJ; Dawu, DW) on blood lipids in middle-aged and elderly individuals. Chinese and English databases were searched, including PubMed, China National Knowledge Infrastructure, Wanfang Database, Chongqing VIP, and Web of Science. A total of 42 randomized controlled trials (RCTs) including 2977 subjects were analyzed. Outcome indicators include total cholesterol (TC), low-density lipoprotein cholesterol (LDL-C), triacylglyceride (TAG), and high-density lipoprotein cholesterol (HDL-C). Summary mean differences (MD) were calculated using pairwise and network meta-analysis with a random-effects model. The results of this study showed that compared to non-exercise intervention (NEI), all six kinds of TCE treatment had some kind of influence on blood lipid indicators, among which WQX and TJQ could improve all four blood lipid indicators, whereas BDJ was effective on three indicators but not on TC. The results of cumulative probability ranking showed that WQX (84.9%, 73.8%, 63.4%, 63.1% to TC, TAG, HDL-C, LDL-C, respectively) was at the top spot being the best intervention, followed by BDJ (55.6%, 83.7%, 68.4%, 56.1%) and TJQ (73.7%, 47.6%, 63.1%, 54.1%). The network meta-analysis of RCTs demonstrates that WQX may be the best TCE treatment for dyslipidemia in middle-aged and elderly individuals.

## 1. Introduction

Blood lipids are an important predictive factor for atherosclerosis and an independent risk factor for coronary heart disease and ischemic stroke, which means that dyslipidemia might lead to many ongoing pathologies in people’s daily life, such as metabolic syndrome and cardiovascular disease [1]. Moreover, a close association between serum lipid levels and the incidence of coronary heart disease has been well proven in middle-aged and elderly people with a probable degeneration of metabolic functions [2]. Lipid profiles have been used to define dyslipidemia, and abnormal serum lipid profiles include changes in four indicators: high total cholesterol (TC), high triacylglyceride (TAG), low high-density lipoprotein cholesterol (HDL-C), and elevated low-density lipoprotein cholesterol (LDL-C) [3]. Therefore, improving the blood lipid indicators of middle-aged and elderly people has become a hot topic that is receiving increased attention at present.

Medicine (e.g., statins, niacin drugs) is widely used in blood lipid improvement [4]. However, with an increasing dose of statins, the occurrence of side effects, such as statin-associated myopathy and liver damage, may increase accordingly [1]. Additionally, statins carry a risk of causing new-onset diabetes, and some experts have even suggested a risk of developing new tumors. This is making patients with elevated blood lipid levels seek complementary and alternative medicine as a lipid-lowering approach. According to the “Nutrition and Health Survey of Chinese Residents”, exercise can decrease the incidence of chronic lipid metabolic syndromes, such as HDL-C and high TAG [5]. Related studies have demonstrated that exercise has the potential to alternate drug treatments, especially in the early phase of dyslipidemia [4]. Thus, it can be seen that appropriate exercise can not only help middle-aged and elderly people to improve their blood lipid indicators but also play a role in physical fitness, which is an ideal treatment without any side effects. However, due to the physical characteristics of middle-aged and elderly people, the mode of exercise cannot be chosen blindly: high-intensity exercise is not suitable, and moderate to low-intensity exercise is the most appropriate option. The common characteristics of traditional Chinese exercises (TCEs) include that they are slow, relaxing, and systematic, and therefore suitable for physically weak patients. TCEs are easy to master in a short time and have few physical demands [2] as they are considered to be a low-risk intervention. Yang Lina et al. (2009) [6] concluded that long-term Taijiquan (TJQ) and Baduanjin (BDJ) exercise can improve the concentration of HDL in blood lipids, reduce the concentration of TAG, TC, and LDL, and reduce the incidence of cardiovascular diseases, such as high blood lipids, in which TCEs were recommended. Therefore, TCE is an exercise mode that can effectively improve blood lipids in middle-aged and elderly people.

TCE might improve the functions of the cardiopulmonary and nervous system as well as balance ability and psychological state [6], which are especially important for middle-aged and older people [7]. The National Fitness Guide issued by the State Sports General Administration of China in 2017 [7] pointed out that TCEs, which include Taijiquan (TJQ), Wuqinxi (WQX), Baduanjin (BDJ), Yijinjing (YJJ), Liuzijue (LZJ), Dawu (DW), Mulan sword, and Martial arts routines, are gentle and safe with emphasis on the combination of meditation and physical activities. Previous studies suggest that TJQ [8], BDJ [9], WQX [10], YJJ [11], LZJ [12], and DW [2] have positive effects on blood lipid indicators. For example, TJQ can improve TAG and HDL levels [8], YJJ can improve TAG, LDL, and HDL levels [11], LZJ can increase HDL levels [12], and DW has a significant effect on improving TC, TAG, and HDL indicators. However, there were differences in the sample sizes, interventions, and inaccurate measurement indicators among these studies, which led to different research results. Through an analysis of these previous studies, it was found that the effect of each TCE on blood lipid indicators was different; in different studies, the effect of the same TCE on blood lipid indicators was also different [6], and comparisons among TCEs have rarely taken place. It is still unclear which TCE is most effective in improving blood lipids among middle-aged and elderly people. To the best of our knowledge, no comprehensive analysis has been undertaken to compare the effect of these TCEs on dyslipidemia treatment. Meta-analyses can generally compare differences in efficacy between two interventions. However, it is difficult to compare the effects among several exercise treatments because of the low number of available head-to-head comparisons. Network meta-analysis (NMA) can overcome this limitation by drawing together direct and indirect comparisons of all available treatment options. Therefore, this systematic review and network meta-analysis compares the effects of TJQ, BDJ, WQX, YJJ, LZJ, and DW on blood lipids, explores the effect of each TCE on the improvement of blood lipid indicators in middle-aged and elderly people, and determines which TCE is the optimal option for middle-aged and elderly individuals with dyslipidemia.

## 2. Materials and Methods

### 2.1. Registration

This study was performed following the PRISMA network statement. The protocol for this study was registered in the International Prospective Register of Systematic Reviews (PROSPERO). The registration number is CRD42020216190.

### 2.2. Data Sources and Search Strategy

The search strategy was created using a combination of medical subject heading (MeSH) terms, keywords, and phrases. The search terms “((Tai Chi) OR (Taijiquan) OR (Yijinjing) OR (Wuqinxi) OR (Five-animal exercise) OR (Liuzijue) OR (Six healing sounds) OR (Yijinjing) OR (Da wu)) AND ((blood lipid) OR (dyslipidemia) OR (dyslipidemias) OR (blood fats)) [Title]” were typed into the databases of PubMed, Web of Science, China National Knowledge Infrastructure (CNKI), and Chongqing VIP in turn, and all the results of these databases were exported to the software EndNote X9. Then, after excluding duplicates, the list of studies was screened using the search terms TC, TAG, HDL-C, and LDL-C in turn at the field of the abstract to screen out studies with eligible outcomes. At the same time, all the studies whose designs or protocols were randomized were included in full-text screenings with the search terms randomized. Studies before 1990 were not included in this study. The search strategy was constructed using the patient, intervention, comparison, outcome, study design (PICOS) framework.

Two researchers (Y.G. and L.Y.) searched the literature and reviewed the title and abstract of each article. The full text of the selected articles was evaluated according to the inclusion and exclusion criteria, and divergent articles were reviewed by a third reviewer (H.H.).

### 2.3. Inclusion and Exclusion Criteria

#### 2.3.1. Inclusion Criteria

The inclusion criteria strategy was defined according to participants, interventions, comparisons, outcomes, and study design (PICOS): (1) Subjects of both sexes, aged 40 years old and over, and with a clinical diagnosis of healthy status, dyslipidemia, or chronic disease were included; (2) the type of study included was RCT comparing the effects of six TCEs on lipids. Experimental groups adopted BDJ, WQX, YJJ, LZJ, DW, or TJQ. The control group was a simple control group with non-exercise intervention (NEI), or routine aerobic exercise (AE), such as walking and jogging, or other specific TCEs; (3) the outcomes included at least one of the four lipid indicators (TC, TAG, HDL, CLDL-C); (4) ethically approved RCTs were included.

#### 2.3.2. Exclusion Criteria

The exclusion criteria included: (1) literature not published in English or Chinese; (2) repetitions of previously published literature; (3) theoretical and review literature; (4) literature with only abstracts but no full text; (5) literature designed for non-randomized controlled studies—for example, before–after studies on the same patient; (6) joint-intervention trials; (7) studies where the experimental subjects were not middle-aged or elderly; (8) studies where the experimental data were not clear, and it was not possible to calculate the average and standard deviation of the outcome indicator; (9) studies with control groups that did not meet our requirements, such as drug control; (10) studies with data errors or missing literature.

### 2.4. Literature Screening and Data Extraction

According to the inclusion and exclusion criteria, a unified method and standardized search and selection were used, with the two authors (Y.G. and L.Y.) conducting searches in turn and independently. The figures and tables for information were produced after the two authors had verified their results. An independent arbitrator (H.H.) resolved any discrepancies in data extraction.

The following data were collected: (1) basic information extracted, including the name of the first author and the year of publication; (2) the demographic characteristics of the subjects, such as sex and age; (3) information regarding study design, such as sample size, interventions, measurement parameters, follow-up duration, and information related to bias risk assessment.

All the measurement parameters of blood lipids were converted to mean differences (MD) on blood lipids following exercise intervention under the international standard system of units. If an included study reported outcomes of different follow-up times or had more than one trial arm, each different follow-up time and trial arm were treated as a separate trial. The Cochrane Handbook for Systematic Review of Interventions provides detailed measures for dealing with such situations—one way to overcome this is to perform a fixed-effect meta-analysis across comparisons within a study, and a random-effects meta-analysis across studies; in practice, the difference between different analyses is likely to be trivial [13].

Since the unit of a certain blood lipid parameter is uniform, the standardized mean difference was not chosen to illustrate the pooled effect. The software STATA^®^ 16 (StataCorp LLC, College Station, TX, USA) was used to analyze the combined effect.

### 2.5. Quality Assessment

The Cochrane Collaboration tool was used to assess the risk of bias. The study quality was assessed and graded independently by two authors (Y.G. and L.Y.) according to the criteria described in The Cochrane Handbook. The risk of bias graph and the risk of bias summary graph were produced using RevMan5.3. If the evaluation results were inconsistent, the issue was resolved following discussion with the third researcher (A.W.).

### 2.6. Statistical Analysis

A set of multivariate meta-analysis programs in STATA^®^ 16 (StataCorp LLC, College Station, TX, USA) software was used to deal with the statistical analysis, draw the net relation diagram of different interventions, and output the table of direct pairwise comparisons of different interventions as well as the forest plot to present the results of network meta-analysis visually. When there was a closed loop, the consistency between direct comparison and indirect comparison was judged by the node-splitting value, and inconsistency was considered to be significant when *p* < 0.05. When there was no closed-loop structure within the interventions, there was no need to make a consistency test. Continuous variables (TC, TAG, HDL-C, and LDL-C) were analyzed by mean differences (MDs) and 95% credible intervals (95%CI). The effectiveness of these six interventions can be ranked by the surface under the cumulative ranking curve (SUCRA). SUCRA values range from 0% to 100%. The higher the SUCRA value, and the closer to 100%, the higher the likelihood that therapy is in the top rank or one of the top ranks.

## 3. Results

### 3.1. Literature Selection

**Figure 1:**
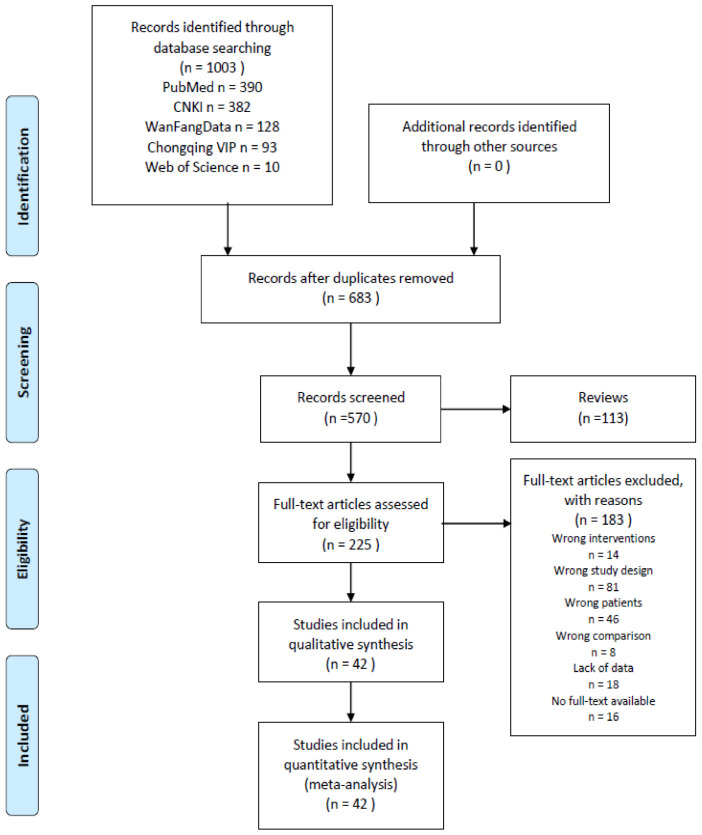
The PRISMA flow diagram of search and study selection; Note: CNKI, China National Knowledge Infrastructure.

In our initial search, we found a total of 1003 articles, of which 320 were duplicates and thus excluded. After deduplication and application of the exclusion criteria, 42 studies [14,15,16,17,18,19,20,21,22,23,24,25,26,27,28,29,30,31,32,33,34,35,36,37,38,39,40,41,42,43,44,45,46,47,48,49,50,51,52,53,54,55] out of the total 1003 studies were included for analysis. The flow diagram can be seen in Figure 1. Based on the information from all the included full texts, the results of data collection and a summary measure of each included study can be seen in Table 1. Figure 1 shows the selection process for the relevant studies.

### 3.2. Characteristics of the Included Studies and Results of Risk of Bias

This review included 42 trials involving 2977 subjects [14,15,16,17,18,19,20,21,22,23,24,25,26,27,28,29,30,31,32,33,34,35,36,37,38,39,40,41,42,43,44,45,46,47,48,49,50,51,52,53,54,55]. All subjects were middle-aged and elderly, with an average age of 55–60 years old. Six exercise interventions (BDJ, WQX, YJJ, LZJ, TJQ, and DW) were included in the current review. The control groups were non-exercise intervention (NEI) and conventional aerobic exercise intervention (AE). There were 41 studies with an experimental period of more than 12 weeks and 1 with less than 12 weeks [31]. Among the included studies, 13 studies compared TJQ with NEI [14,15,16,17,18,19,20,21,22,23,24,25,26], 1 study compared TJQ with AE [24], 8 studies compared BDJ with NEI [28,31,33,34,35,36,37,38], 6 studies compared BDJ with AE [27,29,30,32,36,38], 7 studies compared WQX with NEI [39,40,41,43,44,45,46], 2 studies compared WQX with AE [42,43], 3 studies compared YJJ with NEI [47,48,50], 1 study compared YJJ with AE [49], 2 studies compared LZJ with DW [51,52], 2 studies compared DW with NEI [54,55], and 1 study compared four kinds of exercise (BDJ, WQX, YJJ, and LZJ) [53]. The outcome indicators were TC, TAG, HDL-C, and LDL-C. None of the 42 studies reported any adverse reactions. The main characteristics of each included study are shown in Table 1.

**Figure 2:**
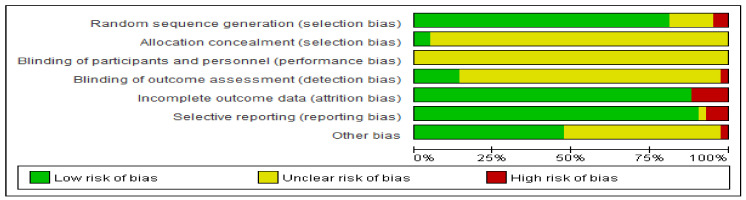
Risk of bias graph for the included RCTs using the bias risk assessment tool recommended in the Cochrane5.1 version of the system review manual.

Of the 42 RCTs, 40 studies did not mention whether the allocation was hidden [14,15,16,17,18,19,20,21,22,24,25,26,27,28,29,30,31,32,33,34,35,37,38,39,40,41,42,43,44,45,46,47,48,49,50,51,52,53,54,55], and none of the included studies mentioned whether the researchers and subjects were double-blinded. Only 6 studies reported blind methods to measure the results in the course of the study [16,18,26,29,41,55], while 5 studies reported the loss of follow-up or withdrawal [26,28,45,49,50] and 3 studies had selective reports [27,28,45]. Other biases are good. The risk of bias graph and summary is shown in Figure 2.

As the intervention methods included in this study were all exercise therapy, most of the included RCTs did not adapt blind methods (patient, care provider, and outcome assessor). In addition, other biases were mainly low-risk bias, so the overall quality of the included literature was high.

### 3.3. The Results of the Network Meta-Analysis

#### 3.3.1. Evidence Network Relationship

**Figure 3:**
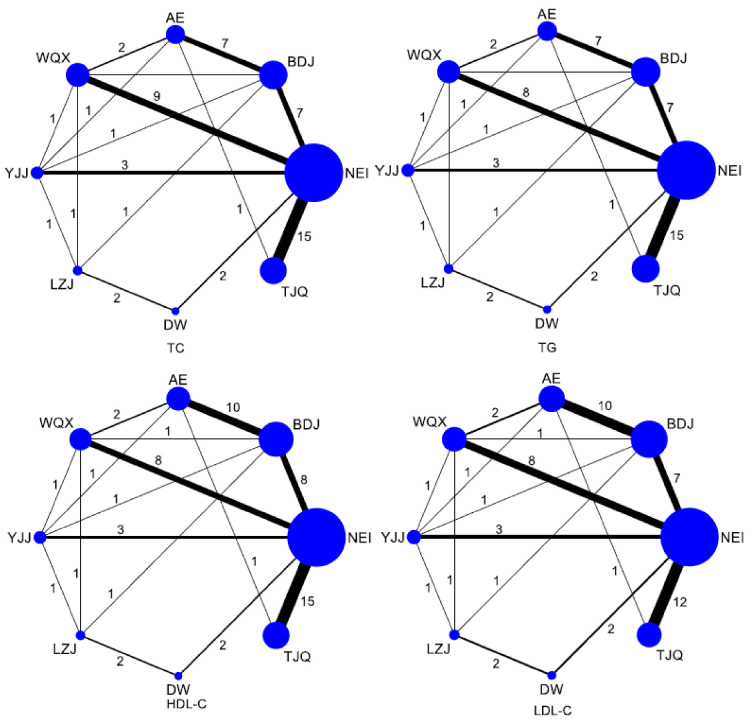
Evidence network diagram of network meta-analysis. Notes: TJQ, Taijiquan; BDJ, Baduanjin; WQX, Wuqinxi; YJJ, Yijinjing; LZJ, Liuzijue; DW, Dawu; NEI, non-exercise intervention; AE, aerobic exercise; The area of the circle, the size of the corresponding intervention study sample size; Nodes represent treatment or intervention; Lines show where direct comparisons exist from one or more studies.

The edges connecting the net point indicate the presence of direct comparison evidence between networks. Numbers in addition to edges indicate the number of independent trials comparing the corresponding pair of treatments head-to-head. Comparisons with more trails have a wider edge. In the network diagram of the effects of eight interventions (including the control group) on the blood lipids of middle-aged and older people (Figure 3), the area of the circle represents the size of the corresponding intervention study sample size. Among all interventions, the order of the sample size was NEI > BDJ > TJQ > WQX > AE > YJJ > LZJ > DW (Figure 3).

#### 3.3.2. TC

**Figure A1:**
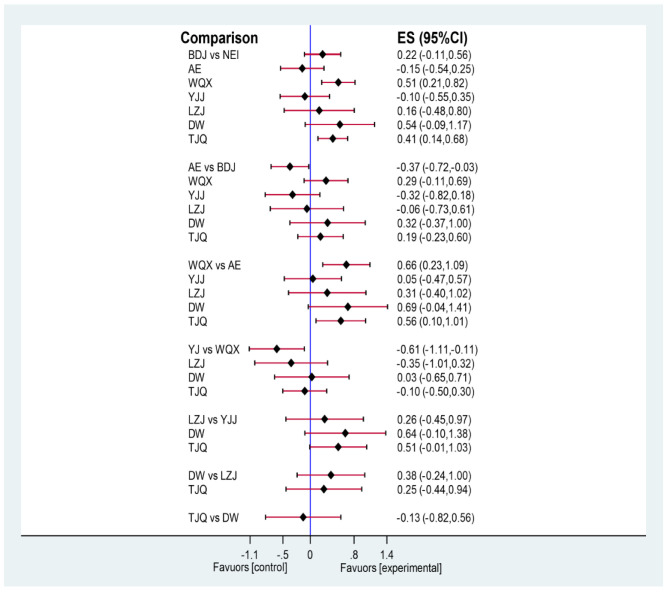
Forest plot of TC. Notes: TJQ, Taijiquan; BDJ, Baduanjin; WQX, Wuqinxi; YJJ, Yijinjing; LZJ, Liuzijue; DW, Dawu; NEI, non-exercise intervention; AE, aerobic exercise; ES, effect size.

**Figure A2:**
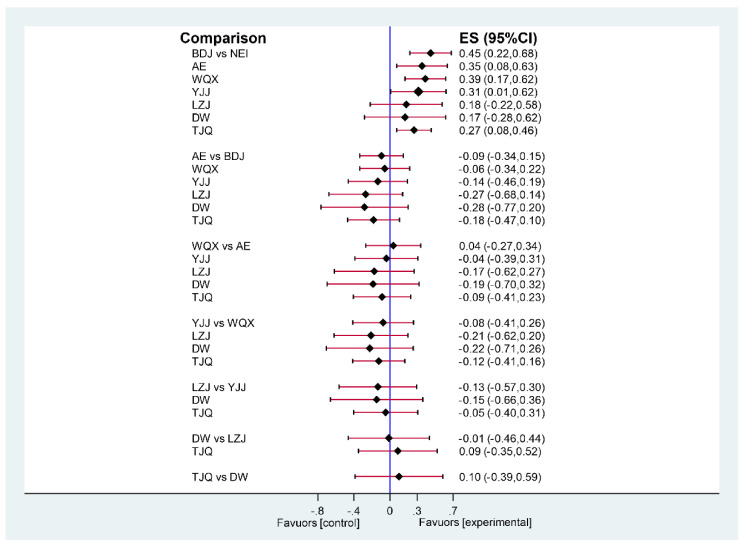
Forest plot of TAG. Notes: TJQ, Taijiquan; BDJ, Baduanjin; WQX, Wuqinxi; YJJ, Yijinjing; LZJ, Liuzijue; DW, Dawu; NEI, non-exercise intervention; AE, aerobic exercise; ES, effect size.

**Figure A3:**
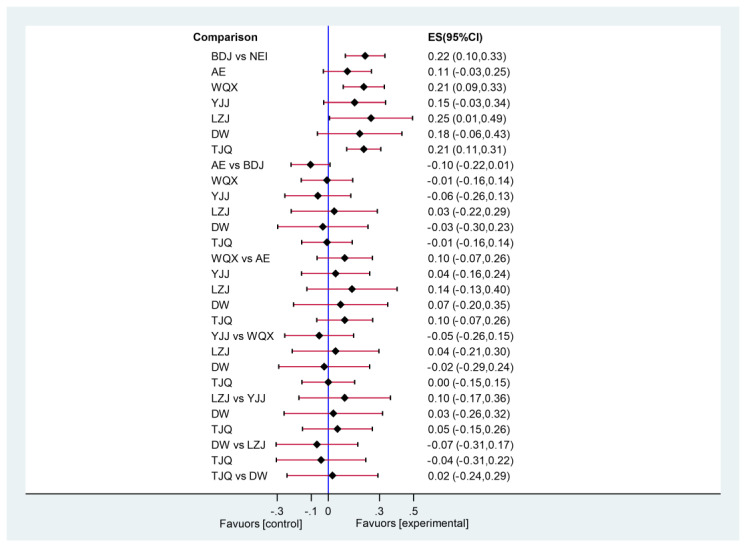
Forest plot of HDL-C. Notes: TJQ, Taijiquan; BDJ, Baduanjin; WQX, Wuqinxi; YJJ, Yijinjing; LZJ, Liuzijue; DW, Dawu; NEI, non-exercise intervention; AE, aerobic exercise; ES, effect size.

**Figure A4:**
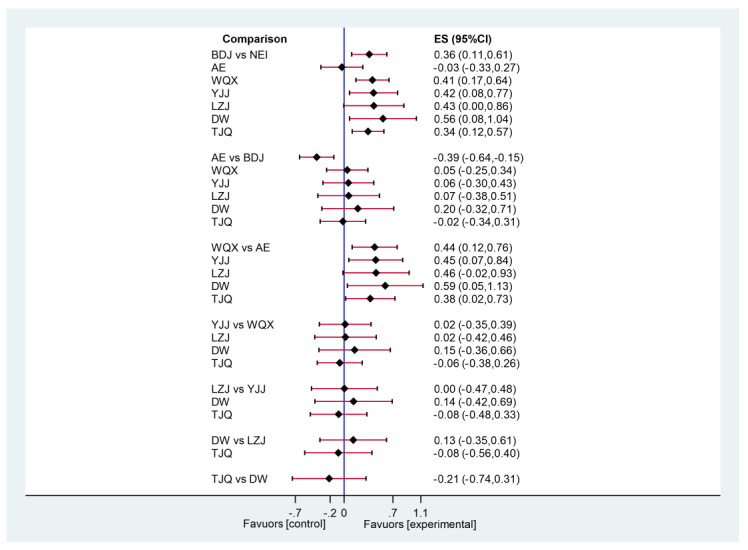
Forest plot of LDL-C. Notes: TJQ, Taijiquan; BDJ, Baduanjin; WQX, Wuqinxi; YJJ, Yijinjing; LZJ, Liuzijue; DW, Dawu; NEI, non-exercise intervention; AE, aerobic exercise; ES, effect size.

Using the classical frequency method with Stata software, traditional direct comparison and indirect comparison were combined at the same time to evaluate all the intervention measures [56]. Network meta-analysis summary plots are shown in Table 2. and Appendix A Figure A1, Figure A2, Figure A3 and Figure A4 (available online).

As shown in Figure 3, a total of 40 studies [14,15,16,17,18,19,20,21,22,23,24,25,26,29,30,31,32,33,34,35,36,37,38,39,40,41,42,43,44,45,46,47,48,49,50,51,52,53,54,55] involving 2816 subjects reported TC levels. Compared with the NEI group, the WQX (MD = 0.51 (0.21–0.82)) and TJQ (MD = 0.41 (0.14, 0.68)) groups were superior in reducing the level of TC (*p* < 0.05). Compared with the AE group, WQX (MD = 0.66 (0.23, 1.09)), TJQ (MD = 0.56 (0.10, 1.01)), and BDJ (MD = 0.37 (0.03, 0.72)) were superior in reducing the level of TC (*p* < 0.05). Compared with the YJJ group, WQX (MD = 0.61 (0.11, 1.11)) was more effective in reducing the TC level (*p* < 0.05). There was no statistical significance in the other groups (Table 2).

#### 3.3.3. TAG

In terms of TAG, there were 39 studies [8,14,15,16,17,18,19,20,21,22,23,24,26,29,30,31,32,33,34,35,36,37,38,39,40,41,42,43,44,46,47,48,49,50,51,52,53,54,55] involving 2712 subjects that were merged for analysis (Figure 3). Compared with the NEI group, the BDJ (MD = 0.45 (0.22, 0.68)), WQX (MD = 0.39 (0.17, 0.62)), YJJ (MD = 0.31 (0.01, 0.62)), TJQ (MD = 0.27 (0.08, 0.46)), and AE (MD = 0.35 (0.08, 0.63)) groups were superior in decreasing the TAG level (*p* < 0.05). There was no statistical significance in the other groups (Table 2).

#### 3.3.4. HDL-C

Regarding HDL-C, a total of 41 studies [8,14,15,16,17,18,19,20,21,22,23,24,26,27,28,29,30,31,32,33,34,35,36,37,38,39,40,41,42,43,44,46,47,48,49,50,51,52,53,54,55] analyzed 2871 subjects (Figure 3). Compared with the NEI group, the LZJ (MD = 0.25 (0.01, 0.49)), BDJ (MD = 0.22 (0.10, 0.33)), TJQ (MD = 0.21 (0.11, 0.31)), and WQX (MD = 0.21 (0.09, 0.33)) groups were superior to the NEI group in improving the level of HDL-C (*p* < 0.05). There was no statistical significance in the other groups (Table 2).

#### 3.3.5. LDL-C

In terms of LDL-C, there were 38 studies [8,14,16,17,18,19,20,21,23,24,27,28,29,30,31,32,34,35,36,37,38,39,40,41,42,43,44,46,47,48,49,50,51,52,53,54,55] involving 2592 subjects (Figure 3). Compared with the NEI or AE group, almost all TCEs showed statistical differences in reducing LDL-C levels. Details are as follows: compared with the NEI group, DW (MD = 0.56 (0.08, 1.04)), LZJ (MD = 0.43 (0.00, 0.86)), YJJ (MD = 0.42 (0.08, 0.77)), WQX (MD = 0.41 (0.17, 0.64)), BDJ (MD = 0.36 (0.11, 0.61)), and TJQ (MD = 0.34 (0.12, 0.57)) were superior in improving the level of LDL-C; compared with the AE group, DW (MD = 0.59 (0.05, 1.13)), YJJ (MD = 0.45 (0.07, 0.84)), WQX (MD = 0.44 (0.12, 0.76)), BDJ (MD = 0.39 (0.15, 0.64)), and TJQ (MD = 0.38 (0.02, 0.73)) were superior in improving the level of LDL-C (*p* < 0.05). There was no statistical significance in the other groups (Table 2).

### 3.4. Intervention Ranking

**Figure 4:**
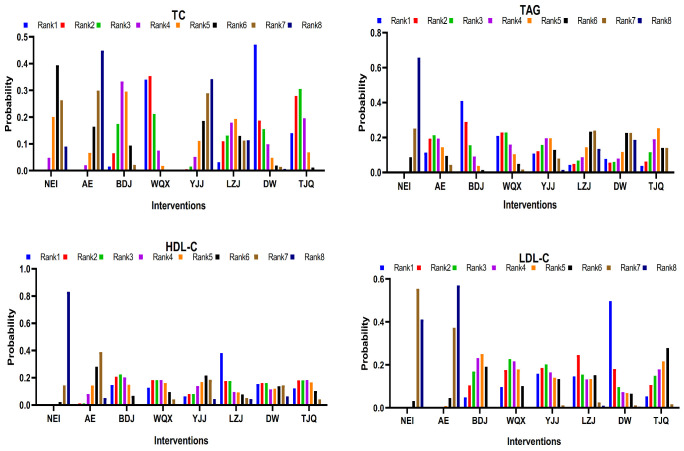
The network rank probabilities. Notes: TJQ, Taijiquan; BDJ, Baduanjin; WQX, Wuqinxi; YJJ, Yijinjing; LZJ, Liuzijue; DW, Dawu; NEI, non-exercise intervention; AE, aerobic exercise; Rank, it refers to the probabilities estimated for each treatment in the network, that is, the chance for each intervention of being ranked as first, second, third, fourth and so on.

**Figure A5:**
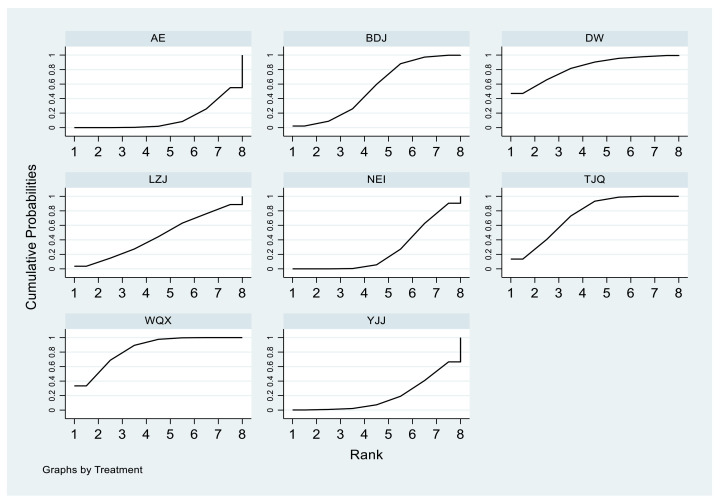
TC. Notes: TJQ, Taijiquan; BDJ, Baduanjin; WQX, Wuqinxi; YJJ, Yijinjing; LZJ, Liuzijue; DW, Dawu; NEI, non-exercise intervention; AE, aerobic exercise.

**Figure A6:**
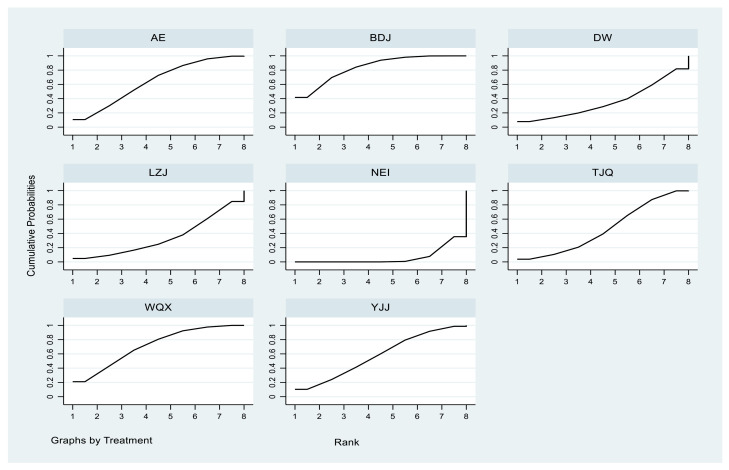
TAG. Notes: TJQ, Taijiquan; BDJ, Baduanjin; WQX, Wuqinxi; YJJ, Yijinjing; LZJ, Liuzijue; DW, Dawu; NEI, non-exercise intervention; AE, aerobic exercise.

**Figure A7:**
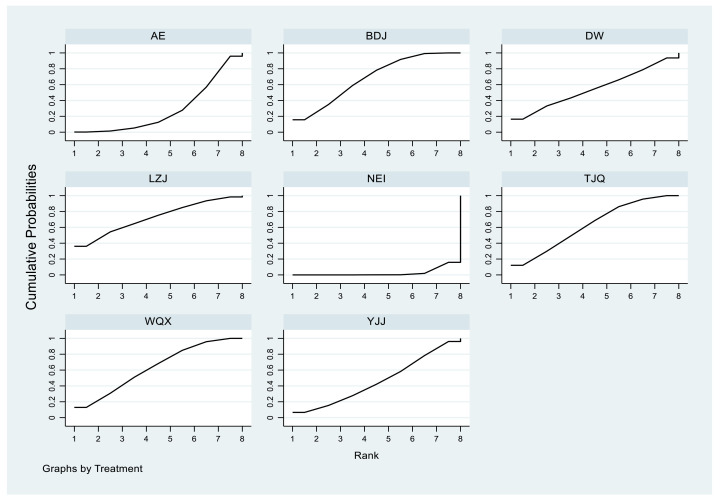
HDL-C. Notes: TJQ, Taijiquan; BDJ, Baduanjin; WQX, Wuqinxi; YJJ, Yijinjing; LZJ, Liuzijue; DW, Dawu; NEI, non-exercise intervention; AE, aerobic exercise.

**Figure A8:**
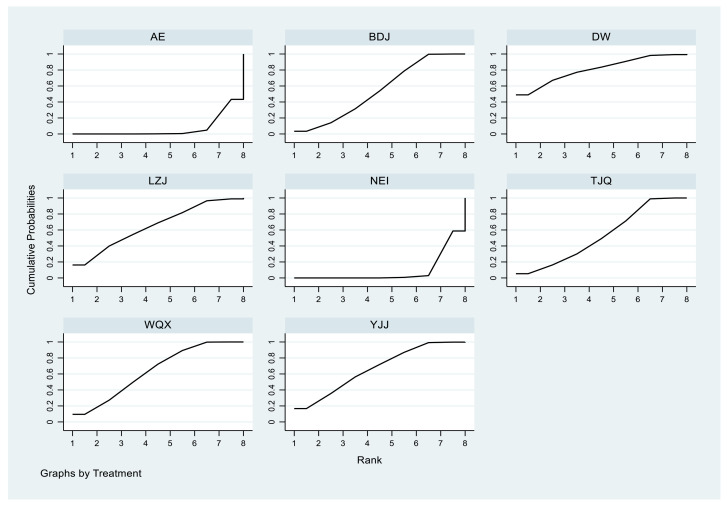
LDL-C. Notes: TJQ, Taijiquan; BDJ, Baduanjin; WQX, Wuqinxi; YJJ, Yijinjing; LZJ, Liuzijue; DW, Dawu; NEI, non-exercise intervention; AE, aerobic exercise.

Figure 4 and Appendix B Table A1, Table A2, Table A3 and Table A4 show the ranking probability of each intervention, Table 3 shows the cumulative probability data of each intervention, and Appendix B Figure A5, Figure A6, Figure A7 and Figure A8 show the surface under the cumulative ranking plots (available online). The ranking probability gram can help researchers to predict the best or worst intervention quickly, but interventions with higher ranking probability are not necessarily the most effective, and there are still many uncertain factors that can interfere with the ranking. If the optimal intervention cannot be obtained, the SUCRA probability gram can help in decision-making [57].

Based on the studies included, the cumulative probability being the best intervention to TC was 84.9%, 81.8%, 73.7%, 55.6%, 44.7%, 27.6%, 18.7%, and 13.0% for WQX, DW, TJQ, BDJ, LZJ, NEI, YJJ, and AE, respectively. The cumulative probability of BDJ, WQX, AE, YJJ, TJQ, DW, LZJ, and NEI being the best intervention of TAG was 83.7%, 73.8%, 63.2%, 56.0%, 47.6%, 35.3%, 33.6%, and 6.9%, respectively. The cumulative probability of LZJ, BDJ, WQX, TJQ, DW, YJJ, AE, and NEI being the best intervention of HDL-C was 72.5%, 68.4%, 63.4%, 63.1%, 55.2%, 46.4%, 28.6%, and 2.6%, respectively. The cumulative probability of DW, YJJ, LZJ, WQX, BDJ, TJQ, NEI, and AE being the best intervention of LDL-C was 80.4%, 65.8%, 64.3%, 63.1%, 56.1%, 54.1%, 9.0%, and 7.2%, respectively (Figure 4, Table 3).

### 3.5. Consistency Analysis

Consistency can be evaluated by node splitting, with each direct comparison being excluded from the network and then estimating the difference between this direct evidence and the indirect evidence from the network. If there are differences, it means that there are inconsistencies, which need to be fitted with an inconsistent model. If there is no difference, it means that there is no inconsistency, and the consistency model is used to fit it [57].

As can be seen from Table 4, all *p* values except AB to TC were greater than 0.05, indicating that there was no obvious inconsistency.

## 4. Discussion

As the General Administration of Sport of China recommends, more and more people are choosing TCEs to exercise at home. This manuscript has been written based on the hypothesis that TECs have a positive effect on the blood lipid parameters of middle-aged and older people. This systematic review included 42 RCTs, with 6 TCEs with 2977 subjects aged 55 to 60 years, providing high-quality evidence of the effect of six kinds of TCE on blood lipids. Maoxing Pan et al. (2019) [58] studied the effect of Qigong on blood lipids in middle-aged and elderly people through a network meta-analysis, but TJQ was not included. Taijiquan is one of the main forms of TCEs, and it is also very popular in China. To the best of our knowledge, at present, this review is the first network meta-analysis comparing the effects of six TCEs.

People’s blood lipid parameters are becoming worse with the development of society. Around the world, over 4000 people die of cardiovascular diseases caused by dyslipidemia every day [59]. Medicine treatments are underused in clinical practice because most doctors worry about their hepatotoxicity and nephrotoxicity [58]. According to a survey of physical examiners in Beijing [59], lifestyle changes play a great role in preventing and treating hypercholesterolemia. TCEs are mind–body exercises that focus on posture, coordination of breathing patterns, and meditation. Movements in TCE are smooth and slow, making this type of exercise safe for middle-aged and older people. In terms of metabolic types, oxidative metabolism, in which fat is oxidized by the body to provide energy, is dominant in TCEs. The positive effects of aerobic exercise on blood lipid parameters have already been verified in many trials enrolling subjects with dyslipidemia [60]. Improvements in lipid levels include lowering the levels of TC, LDL-C, and TAG and increasing the level of HDL-C. Some studies have found that during aerobic exercise, total energy consumption and exercise intensity will affect the improvement of blood lipids and have a positive effect on the improvement of HDL-C and LDL-C [61]. Moderate-intensity aerobic exercise can cause a significant increase in HDL-C, and improvement and reduction in LDL-C may require more intensive aerobic exercise. For middle-aged and elderly individuals, adhering to moderate physical activities can not only delay the senile degenerative changes of various system organs but also maintain relatively high physiological function [62]. Therefore, TCEs can help to improve both physical and psychological conditions of middle-aged and older people [63,64,65].

A total of 42 RCTs were included in this study, including six kinds of TCE and two general controls. Network meta-analysis of direct and indirect evidence showed that six kinds of TCE can effectively improve some blood lipid indicators in middle-aged and elderly individuals. Compared with the NEI group, WQX and TJQ had a significant effect on the decrease in TC, TAG, and LDL and an increase in HDL in middle-aged and elderly people. BDJ had a significant effect on the decrease in TAG and LDL and an increase in HDL in middle-aged and elderly people. YJJ had an obvious effect on the decrease in TAG and LDL in middle-aged and elderly people. LZJ had an obvious effect on the reduction in LDL and can increase the level of HDL in middle-aged and elderly people, while DW had an obvious effect on the reduction in blood LDL in middle-aged and elderly people. Compared with the YJJ group, the level of TC in the WQX group decreased more significantly. We can conclude that all six TCEs are effective in improving LDL-C and are partially effective for other lipid indicators. There are no significant differences in the consistency analysis, which reveals that the statistical model of indirect comparisons is reliable. This may be due to the similar exercise program and outcome indicators among the included studies. Therefore, it can be suggested that patients can choose an appropriate TCE according to their preferences and physical conditions to improve blood lipid parameters.

The ranking results for TC were as follows: WQX > DW > TJQ > BDJ > LZJ > NEI > YJJ > AE. Compared with the NEI group, WQX and TJQ had significant effectiveness, and WQX had the best effect in terms of improving TC. The ranking results for TAG were as follows: BDJ > WQX > AE > YJJ > TJQ > DW > LZJ > NEI. Compared with the NEI group, BDJ, WQX, YJJ, and TJQ had significant effectiveness, and BDJ had the best effect in terms of improving TAG. The ranking results for HDL were as follows: LZJ > BDJ > WQX > TJQ > DW > YJJ > AE > NEI. Compared with the NEI group, LZJ, BDJ, WQX, and TJQ had significant effectiveness, and LZJ had the best effect in terms of improving HDL. The ranking results for LDL were as follows: DW > YJJ > LZJ > WQX > BDJ > TJQ > NEI > AE. Compared with the NEI group, DW, YJJ, LZJ, WQX, BDJ, and TJQ had significant effectiveness, and DW had the best effect in terms of improving LDL. Additionally, statistical factors, such as study design, number of controls, and number of subjects, and many other factors, may have contributed to the difference in outcomes. Although the dominant metabolic patterns of different types of TCE are similar, their activity patterns and training principles are different.

To sum up, based on the comparison with NEI and AE groups, it was found that WQX, TJQ, and BDJ were more effective in improving blood lipids in middle-aged and elderly people. WQX and TJQ had a significant effect on all four indicators of blood lipid indicators. However, BDJ was effective on three indicators of blood lipids, but not TC. Therefore, WQX and TJQ are recommended as appropriate TCE methods for blood lipid control. The results of cumulative probability ranking showed that the SUCRA value of WQX was the highest, while the PrBest value, followed by BDJ and TJQ, indicated that WQX may be the most effective TCE to improve the blood lipid status of middle-aged and elderly people. Therefore, two methods (WQX and TJQ) are recommended as TCE methods for blood lipid control, of which WQX is recommended as the best choice.

The influential mechanism of WQX on blood lipids may be that it emphasizes the control of respiration and regulation of the mind during exercise [66], which not only improves the function of the respiratory system but also accelerates blood circulation [67]. By controlling breathing, the balance of the autonomic nerve can be regulated [68], and the level of substance metabolism can be regulated, and thus, the disorder of lipid metabolism can be improved [39]. The essence of mind regulation is to dominate and exercise the motor nervous system and autonomic nervous system, regulate hormone-sensitive lipase by increasing the activity of the sympathetic nervous system, and finally regulate lipid metabolism [39]. The action mechanism of TJQ may be that its action plays a natural “massage” effect on vascular smooth muscle, promoting rhythmic contraction and relaxation of blood vessels [21], gradually scouring and eliminating the deposition of cholesterol and cholesterol esters on the blood vessel wall; furthermore, TJQ exercise helps to eliminate the anxiety and tension of practitioners, reduce the tension of sympathetic vasoconstrictor nerves, reduce the release of norepinephrine in its terminals [69], and improve the excitability of sympathetic vasoconstrictor nerves. However, the specific mechanism of these three kinds of TCE on improving blood lipids is not clear, with a noted lack of high-quality research at the cellular and molecular level, and thus, further research is needed.

There were some limitations in this network meta-analysis. Small sample sizes and large sex differences existed in some RCTs. There was a different number of RCTs included in different interventions, which may have had a certain impact on the evaluation of the intervention. Most of the RCTs were conducted in China and published in Chinese, which may affect the results. The differences between analyzed populations (middle-aged and elderly) were not reported. Most RCTs did not clearly report whether a random, double-blind method was used. The research did not discuss the adaptation time, size, and actual needs of each TCE.

In follow-up studies, after the emergence of more multicenter, standardized, and high-quality research using larger samples, we can make use of the advantages of network meta-analysis to further study the impact of TCEs on blood lipids among middle-aged and elderly people from all aspects. More research and attention should be paid to the time for adaptation, magnitude, and practical needs of each TCE, as well as the specific mechanism and the best training program for each TCE.

## 5. Conclusions

Our network meta-analysis suggested that six kinds of TCE (WQX, BDJ, TJQ, YJJ, LZJ, and DW) are all effective in partially improving blood lipid indicators among middle-aged and elderly people, while WQX and TJQ can be effective for all four blood lipid indicators and seem to be recommended as the most appropriate way for the elderly to exercise. WQX, BDJ, LZJ, and DW might be the most effective, respectively, for improving TC, TAG, HDL-C, and LDL-C among the six kinds of TCE. According to a comprehensive ranking, WQX had the best effect on improving blood lipids. However, due to the limitations of this study, follow-up studies need to classify and explore the disease status of middle-aged and elderly people and further study the effects of exercise duration and frequency on blood lipid parameters. Furthermore, high-quality clinical trials are needed in the future to strengthen the supportive evidence.

## Figures and Tables

**Table 1:** The characteristics of the included studies.

Study ID	Subjects	Sample Size (Female/Male)	Age	Interventions	Duration	Frequency	Outcomes
Liu, 2007	Dyslipidemia	T:30(16/14) C:30(15/15)	T:65.34 C:65.64	TJQ vs. NEI	16 weeks	5 times per week for 60 min	① ② ③ ④
Wang Z S, 1999 (1)	Healthy	T:28(0/28) C:25(0/25)	T:68.16 C:67.28	TJQ vs. NEI	48 weeks	N\A	① ② ③
Wang Z S, 1999 (2)	Healthy	T:28(0/28) C:25(0/25)	T:68.16 C:67.28	TJQ vs. NEI	96 weeks	N\A	① ② ③
Cui, 2019(1)	Healthy	T:30(N\A) C:30(N\A)	T:54.67 C:55.37	TJQ vs. NEI	96 weeks	3 times per week for 60 min	① ② ③ ④
Cui, 2019(2)	Healthy	T:30(N\A) C:30(N\A)	T:64.72 C:65.91	TJQ vs. NEI	96 weeks	3 times per week for 60 min	① ② ③ ④
Yin, 2019	Chronic disease	T:26(20/6) C:20(16/4)	T:51.88 C:53.10	TJQ vs. NEI	64 weeks	5 times per week for 60 min	① ② ③ ④
Shen, 2018	Chronic disease	T:23(18/5) C:15(12/3)	T:66.57 C:66.27	TJQ vs. NEI	96 weeks	6 times per week for 60 min	① ② ③ ④
Zhao, 2017	Chronic disease	T:8(0/8) C:8(0/8)	T:54.75 C:52.38	TJQ vs. NEI	64 weeks	7 times per week for 60 min	① ② ③ ④
Jiang R K, 2007	Chronic disease	T:18(12/6) C:18(11/7)	T:57.20 C:55.00	TJQ vs. NEI	80 weeks	1.5 times per week for 38 min	① ② ③ ④
Chen, 2013	Chronic disease	T:32(11/21) C:28(10/18)	T:69.30 C:68.70	TJQ vs. NEI	12 weeks	4 times per week for 60 min	① ② ③ ④
Lan, 2008	Dyslipidemia	T:28(16/12) C:25(13/12)	T:52.80 C:50.10	TJQ vs. NEI	48 weeks	3 times per week for 60 min	① ② ③
Tsai, 2003	Healthy	T:37(19/18) C:39(19/20)	T:50.50 C:51.60	TJQ vs. NEI	48 weeks	3 times per week for 50 min	① ② ③ ④
Thomas, 2005 (1)	Healthy	T:64(30/34) C:65(30/35)	T:68.90 C:69.10	TJQ vs. AE	48 weeks	3 times per week for 60 min	① ② ③ ④
Thomas, 2005 (2)	Healthy	T:64(30/34) C:78(34/44)	T:68.90 C:69.00	TJQ vs. NEI	48 weeks	3 times per week for 60 min	① ② ③ ④
Zhang Y, 2008	Chronic disease	T:10(10/0) C:10(10/0)	T:57.40 C:57.40	TJQ vs. NEI	56 weeks	5 times per week for 60 min	① ② ③ ④
Chen, 2009	Chronic disease	T:50(31/19) C:44(20/24)	T:59.10 C:58.30	TJQ vs. NEI	48 weeks	3 times per week for 60 min	① ② ③
Liu, 2005 (1)	Healthy	T:20(N\A) C:22(N\A)	T:51.88 C:60.00	BDJ vs. AE	12 weeks	14 times per week for 20 min	③ ④
Liu, 2005 (2)	Chronic disease	T:21(N\A) C:22(N\A)	T:60.00 C:60.00	BDJ vs. AE	12 weeks	14 times per week for 20 min	③ ④
Liu, 2005 (3)	Chronic disease	T:10(N\A) C:10(N\A)	T:60.00 C:60.00	BDJ vs. AE	12 weeks	14 times per week for 20 min	③ ④
Si, 2009	Healthy	T:27(27/0) C:27(27/0)	T:46.20 C:48.50	BDJ vs. NEI	12 weeks	14 times per week for 15 min	③ ④
Liang, 2014	Chronic disease	T:30(10/20) C:30(12/18)	T:54.80 C:55.70	BDJ vs. AE	24 weeks	10 times per week for 20 min	① ② ③ ④
Liu, 2006 (1)	Healthy	T:20(N\A) C:22(N\A)	T: N\A C: N\A	BDJ vs. AE	12 weeks	14 times per week for 30 min	① ② ③ ④
Liu, 2006 (2)	Chronic disease	T:21(N\A) C:20(N\A)	T: N\A C: N\A	BDJ vs. AE	12 weeks	14 times per week for 30 min	① ② ③ ④
Liu, 2006 (3)	Chronic disease	T:6(N\A) C:6(N\A)	T: N\A C: N\A	BDJ vs. AE	12 weeks	14 times per week for 30 min	① ② ③ ④
Zhang, 2008	Dyslipidemia	T:13(13/0) C:23(23/0)	T:41.00 C:40.96	BDJ vs. NEI	10 weeks	5 times per week for 55 min	① ② ③ ④
Sun G, 2004	Healthy	T:19(0/19) C:17(0/17)	T:65.74 C:65.29	BDJ vs. AE	12 weeks	5 times per week for 45 min	① ② ③ ④
Wang, 2007	Chronic disease	T:40(12/28) C:39(14/25)	T:57.80 C:56.50	BDJ vs. NEI	24 weeks	14 times per week for 60 min	① ② ③
Yang, 2012	Chronic disease	T:29(20/9) C:30(15/15)	T:60.83 C:58.10	BDJ vs. NEI	24 weeks	21 times per week for 60 min	① ② ③ ④
Miao, 2009	Dyslipidemia	T:25(13/12) C:24(12/12)	T:63.32 C:63.68	BDJ vs. NEI	72 weeks	6 times per week for 55 min	① ② ③ ④
Fang, 2014 (1)	Chronic disease	T:30(14/16) C:30(13/17)	T:56.62 C:57.13	BDJ vs. NEI	12 weeks	10 times per week for 30 min	① ② ③ ④
Fang, 2014 (2)	Chronic disease	T:30(14/16) C:29(14/15)	T:56.62 C:58.24	BDJ vs. AE	12 weeks	10 times per week for 30 min	① ② ③ ④
Sun, 2018	Healthy	T:5(N\A) C:9(N\A)	T:53.40 C:58.40	BDJ vs. NEI	16 weeks	5 times per week for 60 min	① ② ③ ④
Sun G, 2008 (1)	Healthy	T:19(0/19) C:20(0/20)	T:65.70 C:64.80	BDJ vs. NEI	12 weeks	5 times per week for 45 min	① ② ③ ④
Sun G, 2008 (2)	Healthy	T:19(0/19) C:17(0/17)	T:65.70 C:65.23	BDJ vs. AE	12 weeks	5 times per week for 45 min	① ② ③ ④
Yu, 2008 (1)	Healthy	T:12(0/12) C:15(0/15)	T: N\A C: N\A	WQX vs. NEI	24 weeks	4 times per week for 45 min	① ② ③ ④
Yu, 2008 (2)	Healthy	T:34(34/0) C:35(35/0)	T: N\A C: N\A	WQX vs. NEI	24 weeks	4 times per week for 45 min	① ② ③ ④
Yuan, 2011 (1)	Healthy	T:57(38/19) C:53(36/17)	T:61.40 C:62.11	WQX vs. NEI	12 weeks	5 times per week for 60 min	① ② ③ ④
Yuan, 2011 (2)	Healthy	T:54(37/17) C:47(31/16)	T:67.24 C:67.42	WQX vs. NEI	12 weeks	5 times per week for 60 min	① ② ③ ④
Yan, 2009	Chronic disease	T:31(8/23) C:31(9/22)	T: N\A C: N\A	WQX vs. NEI	24 weeks	7 times per week for 60 min	① ② ③ ④
Li, 2009	Dyslipidemia	T:33(14/19) C:33(12/21)	T:58.67 C:56.47	WQX vs. AE	16 weeks	7 times per week for 30 min	① ② ③ ④
Ru, 2013 (1)	Healthy	T:19(N\A) C:20(N\A)	T:65.60 C:64.90	WQX vs. NEI	24 weeks	5 times per week for 45 min	① ② ③ ④
Ru, 2013 (2)	Healthy	T:19(N\A) C:18(N\A)	T:65.60 C:65.50	WQX vs. AE	24 weeks	5 times per week for 45 min	① ② ③ ④
Sha, 2010	Healthy	T:40 (40/0) C:40(40/0)	T:57.78 C:57.68	WQX vs. NEI	20 weeks	5 times per week for 60 min	① ② ③ ④
Shen, 2015	Healthy	T:48(N\A) C:56(N\A)	T: N\A C: N\A	WQX vs. NEI	48 weeks	5 times per week for 60 min	①
Sun H M, 2015	Healthy	T:15(0/15) C:15(0/15)	T: N\A C: N\A	WQX vs. NEI	24 weeks	5 times per week for 45 min	① ② ③ ④
Meng, 2017	Chronic disease	T:12(3/9) C:12(2/10)	T: N\A C: N\A	YJJ vs. NEI	12 weeks	3 times per week for 60 min	① ② ③ ④
Su, 2012	Healthy	T:35(35/0) C:35(35/0)	T: N\A C: N\A	YJJ vs. NEI	12 weeks	5 times per week for 60 min	① ② ③ ④
Yuan M, 2014	Dyslipidemia	T:30(14/16) C:30(15/15)	T:50.93 C:48.79	YJJ vs. AE	24 weeks	5 times per week for 30 min	① ② ③ ④
Liu, 2010	Healthy	T:32(32/0) C:30(30/0)	T: N\A C: N\A	YJJ vs. NEI	24 weeks	6 times per week for 45 min	① ② ③ ④
Wang S Y, 2018	Healthy	T:25(0/25) C:25(0/25)	T:60.00 C:60.00	LZJ vs. DW	24 weeks	3 times per week for 60 min	① ② ③ ④
Cui M, 2018	Healthy	T:15(15/0) C:15(15/0)	T:57.31 C:59.91	LZJ vs. DW	24 weeks	3 times per week for 45 min	① ② ③ ④
Wei, 2007 (1)	Healthy	T:11(N\A) C:11(N\A)	T:60.00 C:60.00	BDJ vs. YJJ	12 weeks	5 times per week for 60 min	① ② ③ ④
Wei, 2007 (2)	Healthy	T:11(N\A) C:11 (N\A)	T:60.00 C:60.00	BDJ vs. WQX	12 weeks	5 times per week for 60 min	① ② ③ ④
Wei, 2007 (3)	Healthy	T:11(N\A) C:11 (N\A)	T:60.00 C:60.00	BDJ vs. LZJ	12 weeks	5 times per week for 60 min	① ② ③ ④
Wei, 2007 (4)	Healthy	T:11(N\A) C:11 (N\A)	T:60.00 C:60.00	WQX vs. YJJ	12 weeks	5 times per week for 60 min	① ② ③ ④
Wei, 2007 (5)	Healthy	T:11(N\A) C:11 (N\A)	T:60.00 C:60.00	WQX vs. LZJ	12 weeks	5 times per week for 60 min	① ② ③ ④
Wei, 2007 (6)	Healthy	T:11(N\A) C:11 (N\A)	T:60.00 C:60.00	YJJ vs. LZJ	12 weeks	5 times per week for 60 min	① ② ③ ④
Han, 2017	Healthy	T:15(0/15) C:15(0/15)	T:61.12 C:61.49	DW vs. NEI	24 weeks	3 times per week for 55 min	① ② ③ ④
Zhu, 2017	Healthy	T:15(15/0) C:15(15/0)	T:59.91 C:60.02	DW vs. NEI	24 weeks	3 times per week for 60 min	① ② ③ ④

Notes: T, treatment group; C, control group; TJQ, Taijiquan; BDJ, Baduanjin; WQX, Wuqinxi; YJJ, Yijinjing; LZJ, Liuzijue; DW, Dawu; NEI, non-exercise intervention; AE, aerobic exercise; N\A, not available; ①, total cholesterol; ②, triacylglyceride; ③, high-density lipoprotein cholesterol; ④, low-density lipoprotein cholesterol.

**Table 2:** Comparative analysis of total effective rate.

Items	TC	TAG	HDL-C	LDL-C	
WQX	TJQ	0.10(−0.30, 0.50)	0.12(−0.16, 0.41)	0.00(−0.15, 0.15)	0.06(−0.26, 0.38)
	BDJ	0.29(−0.11, 0.69)	−0.06(−0.34, 0.22)	−0.01(−0.16, 0.14)	0.05(−0.25, 0.34)
	DW	−0.03(−0.71, 0.65)	0.22(−0.26, 0.71)	0.02(−0.24, 0.29)	−0.15(−0.66, 0.36)
	LZJ	0.35(−0.32, 1.01)	0.21(−0.20, 0.62)	−0.04(−0.30, 0.13)	−0.02(−0.46, 0.42)
	YJJ	0.61(0.11, 1.11) *	0.08(−0.26, 0.41)	0.05(−0.15, 0.26)	−0.02(−0.39, 0.35)
	AE	0.66(0.23, 1.09) *	0.04(−0.27, 0.34)	0.10(−0.16, 0.24)	0.44(0.12, 0.76) *
	NEI	0.51(0.21, 0.82) *	0.39(0.17, 0.62) *	0.21(0.09, 0.33) *	0.41(0.17, 0.64) *
TJQ	BDJ	0.19(−0.23, 0.60)	−0.18(−0.47, 0.10)	−0.01(−0.16, 0.14)	−0.02(−0.34, 0.31)
	DW	−0.1(−0.82, 0.56)	0.10(−0.39, 0.59)	0.02(−0.24, 0.29)	−0.21(−0.74, 0.31)
	LZJ	0.25(−0.44, 0.94)	0.09(−0.35, 0.52)	−0.04(−0.31, 0.22)	−0.08(−0.56, 0.40)
	YJJ	0.51(−0.01, 1.03)	−0.05(−0.40, 0.31)	0.05(−0.15, 0.26)	−0.08(−0.48, 0.33)
	AE	0.56(0.10, 1.01) *	−0.09(−0.41, 0.23)	0.10(−0.07, 0.26)	0.38(0.02, 0.73) *
	NEI	0.41(0.14, 0.68) *	0.27(0.08, 0.46) *	0.21(0.11, 0.31) *	0.34(0.12, 0.57) *
BDJ	DW	−0.32(−1.00, 0.37)	0.28(−0.20, 0.77)	0.03(−0.23, 0.30)	−0.20(−0.71, 0.32)
	LZJ	0.06(−0.61, 0.73)	0.27(−0.14, 0.68)	−0.03(−0.29, 0.22)	−0.07(−0.51, 0.38)
	YJJ	0.32(−0.18, 0.82)	0.14(−0.19, 0.46)	0.06(−0.13, 0.26)	−0.06(−0.43, 0.30)
	AE	0.37(0.03, 0.72) *	0.09(−0.15, 0.34)	0.10(−0.01, 0.22)	0.39(0.15, 0.64) *
	NEI	0.22(−0.11, 0.56)	0.45(0.22, 0.68) *	0.22(0.10, 0.33) *	0.36(0.11, 0.61) *
DW	LZJ	0.38(−0.24, 1.00)	−0.01(−0.46, 0.44)	−0.07(−0.31, 0.17)	0.13(−0.35, 0.61)
	YJJ	0.64(−0.10, 1.38)	−0.15(−0.66, 0.36)	0.03(−0.26, 0.32)	0.14(−0.42, 0.69)
	AE	0.69(−0.04, 1.41)	−0.19(−0.70, 0.32)	0.07(−0.20, 0.35)	0.59(0.05, 1.13) *
	NEI	0.54(−0.09, 1.17)	0.17(−0.28, 0.62)	0.18(−0.06, 0.43)	0.56(0.08, 1.04) *
LZJ	YJJ	0.26(−0.45, 0.97)	−0.13(−0.57, 0.30)	0.10(−0.17, 0.36)	0.00(−0.47, 0.48)
	AE	0.31(−0.40, 1.02)	−0.17(−0.62, 0.27)	0.14(−0.13, 0.40)	0.46(−0.02, 0.93)
	NEI	0.16(−0.48, 0.80)	0.18(−0.22, 0.58)	0.25(0.01, 0.49) *	0.43(0.00, 0.86) *
YJJ	AE	0.05(−0.47, 0.57)	−0.04(−0.39, 0.31)	0.04(−0.16, 0.24)	0.45(0.07, 0.84) *
	NEI	−0.10(−0.55, 0.35)	0.31(0.01, 0.62) *	0.15(−0.03, 0.34)	0.42(0.08, 0.77) *
AE	NEI	−0.15(−0.54, 0.25)	0.35(0.08, 0.63) *	0.11(−0.03, 0.25)	−0.03(−0.33, 0.27)

Notes: TJQ, Taijiquan; BDJ, Baduanjin; WQX, Wuqinxi; YJJ, Yijinjing; LZJ, Liuzijue; DW, Dawu; NEI, non-exercise intervention; AE, aerobic exercise; This table is a combination of direct and indirect evidence in this network of meta-analysis. The data represent the comparison of outcomes between two interventions. * Data are mean differences (95% CI) of combined effect, *p* < 0.05.

**Table 3:** A network league table based on the network meta-analysis from data.

Treatments/Outcomes	SUCRA	PrBest	Mean Rank
TC			
WQX	84.9	36.8	2.1
DW	81.8	44.9	2.3
TJQ	73.7	12.8	2.8
BDJ	55.6	2.2	4.1
LZJ	44.7	3.1	4.9
NEI	27.6	0.0	6.1
YJJ	18.7	0.2	6.7
AE	13.0	0.0	7.1
TAG			
BDJ	83.7	39.5	2.1
WQX	73.8	23.3	2.8
AE	63.2	11.1	3.6
YJJ	56.0	11.1	4.1
TJQ	47.6	3.5	4.7
DW	35.3	7.9	5.5
LZJ	33.6	3.6	5.6
NEI	6.9	0	7.5
HDL-C			
LZJ	72.5	36.1	2.9
BDJ	68.4	15.7	3.2
WQX	63.4	12.9	3.6
TJQ	63.1	12.1	3.6
DW	55.2	16.4	4.1
YJJ	46.4	6.6	4.8
AE	28.6	0.2	6.0
NEI	2.6	0.0	7.8
LDL-C			
DW	80.4	47.9	2.4
YJJ	65.8	16.1	3.4
LZJ	64.3	14.2	3.5
WQX	63.1	10.4	3.6
BDJ	56.1	5.6	4.1
TJQ	54.1	5.8	4.2
NEI	9.0	0.0	7.4
AE	7.2	0.0	7.5

Notes: TJQ, Taijiquan; BDJ, Baduanjin; WQX, Wuqinxi; YJJ, Yijinjing; LZJ, Liuzijue; DW, Dawu; NEI, non-exercise intervention; AE, aerobic exercise; SUCRA, the surface under the cumulative ranking; PrBest, The probability that the treatment becomes the best treatment; Mean Rank, the ranking of the treatment measures.

**Table 4:** Inconsistency test between direct and indirect treatment comparisons in mixed treatment comparison.

Side	Direct		Indirect		Difference		*p* > |z|
	Coef.	Std. Err.	Coef	Std. Err.	Coef.	Std. Err.	
TC							
A B	−0.09	0.20	0.78	0.27	−0.87	0.33	0.01
A D	0.60	0.18	0.23	0.32	0.38	0.36	0.30
A E	0.03	0.3	−0.26	0.34	0.30	0.47	0.53
A G	0.62	0.40	0.39	0.56	0.24	0.69	0.73
A H	0.46	0.14	−0.26	0.53	0.72	0.55	0.19
B C	−0.56	0.20	0.12	0.32	−0.68	0.38	0.07
B D	−0.05	0.64	0.33	0.22	−0.37	0.67	0.58
B E	−0.75	0.66	−0.25	0.28	−0.50	0.71	0.48
B F	−0.39	0.67	0.05	0.40	−0.44	0.78	0.57
C D	0.35	0.37	0.82	0.27	−0.47	0.46	0.31
C E	0.06	0.52	0.04	0.31	0.02	0.61	0.98
C H	0.67	0.45	0.72	0.26	−0.72	0.55	0.19
D E	−0.70	0.65	−0.60	0.28	−0.10	0.71	0.88
D F	−0.34	0.66	−0.35	0.40	0.01	0.77	0.99
E F	0.36	0.68	0.22	0.43	0.14	0.80	0.87
F G	0.31	0.38	0.54	0.57	−0.24	0.69	0.73
TAG							
A B	0.41	0.15	0.51	0.19	−0.10	0.24	0.68
A D	0.44	0.14	0.29	0.21	0.15	0.25	0.57
A E	0.25	0.24	0.37	0.21	−0.11	0.32	0.73
A G	0.13	0.30	0.23	0.38	−0.11	0.48	0.82
A H	0.28	0.10	0.13	0.37	0.15	0.39	0.70
B C	−0.10	0.15	−0.09	0.23	−0.01	0.27	0.98
B D	−0.20	0.38	−0.12	0.19	−0.06	0.42	0.90
B E	−0.18	0.38	−0.12	0.19	−0.06	0.42	0.90
B F	−0.31	0.38	−0.25	0.26	−0.06	0.46	0.90
C D	0.05	0.28	0.03	0.19	0.02	0.34	0.97
C E	0.05	0.35	−0.07	0.21	0.12	0.41	0.76
C H	−0.20	0.34	−0.05	0.19	−0.15	0.39	0.70
D E	0.02	0.38	−0.10	0.20	0.12	0.43	0.77
D F	−0.11	0.38	−0.26	0.26	0.15	0.46	0.74
E F	−0.13	0.38	−0.14	0.28	0.01	0.47	0.99
F G	0.03	0.29	−0.08	0.38	0.11	0.48	0.82
HDL-C							
A B	0.18	0.07	0.30	0.11	−0.12	0.13	0.35
A D	0.21	0.07	0.22	0.13	−0.01	0.15	0.93
A E	0.28	0.13	0.03	0.13	0.24	0.19	0.20
A G	0.13	0.16	0.28	0.21	−0.14	0.27	0.59
A H	0.22	0.05	0.09	0.20	0.12	0.21	0.56
B C	−0.12	0.07	−0.04	0.13	−0.09	0.14	0.54
B D	−0.01	0.29	−0.01	0.08	−0.00	0.30	0.48
B E	−0.24	0.27	−0.03	0.11	−0.21	0.30	0.48
B F	0.01	0.26	0.04	0.15	−0.03	0.30	0.91
C D	0.05	0.15	0.12	0.10	−0.74	0.18	0.69
C E	0.07	0.19	0.32	0.12	0.04	0.23	0.87
C H	0.09	0.19	0.12	0.09	−0.12	0.21	0.56
D E	−0.23	0.26	−0.22	0.11	−0.21	0.29	0.47
D F	0.02	0.25	0.05	0.15	−0.03	0.30	0.42
E F	0.25	0.24	0.02	0.17	0.23	0.29	0.42
F G	−0.02	0.14	−0.17	0.22	0.14	0.27	0.59
LDL-C							
A B	0.20	0.16	0.59	0.20	−0.39	0.26	0.13
A D	0.53	0.14	0.10	0.22	0.42	0.26	0.11
A E	0.26	0.27	0.55	0.23	−0.29	0.36	0.42
A G	0.49	0.31	0.67	0.40	−0.18	0.51	0.73
A H	0.38	0.12	−0.11	0.40	0.49	0.42	0.25
B C	−0.52	0.14	0.00	0.24	−0.52	0.28	0.06
B D	−0.08	0.40	0.07	0.17	−0.15	0.43	0.74
B E	0.25	0.41	0.01	0.21	0.24	0.47	0.61
B F	0.12	0.41	0.04	0.28	0.08	0.49	0.87
C D	0.26	0.30	0.52	0.20	−0.26	0.36	0.47
C E	0.22	0.40	0.53	0.23	−0.31	0.46	0.49
C H	0.09	0.37	0.49	0.21	−0.49	0.42	0.25
D E	0.20	0.40	−0.07	0.21	0.40	0.46	0.38
D F	0.20	0.40	−0.06	0.30	−0.20	0.51	0.69
E F	−0.13	0.41	0.07	0.30	−0.20	0.51	0.69
F G	0.20	0.31	0.02	0.40	0.18	0.51	0.73

Notes: Coef., coefficient; Std. Err., standard error; A, non-exercise intervention; B, Baduanjin; C, aerobic exercise; D, Wuqinxi; E, Yijinjing; F, Liuzijue; G, Dawu; H, Taijiquan; *p* > 0.05, there is no inconsistency.

## Data Availability

Detailed data supporting reported results can be found at Figure A1, Figure A2, Figure A3 and Figure A4 in Appendix A and Table A1, Table A2, Table A3 and Table A4 Appendix B.

## Abbreviations

TCE	traditional Chinese exercise;
TJQ	Taijiquan;
WQX	Wuqinxi;
LZJ	Liuzijue;
YJJ	Yijinjing;
DW	Dawu;
NEI	non-exercise intervention;
AE	aerobic exercise;
TC	total cholesterol;
TAG	triacylglyceride;
HDL-C	high-density lipoprotein cholesterol;
LDL-C	low-density lipoprotein cholesterol;
RCT	randomized controlled trials;
MD	mean differences;
SUCRA	the surface under the cumulative ranking;
NMA	network meta-analysis;
CNKI	National Knowledge Infrastructure.

## Appendix A

Figure A1, Figure A2, Figure A3 and Figure A4. Network meta-analysis summary plot.

## Appendix B

Figure A5, Figure A6, Figure A7 and Figure A8. The surface under the cumulative ranking plots based on the cumulative probabilities of the interventions.

Table A1, Table A2, Table A3 and Table A4 Different ranking probabilities of Interventions.

**Table A1:** TC.

	Rank1	Rank2	Rank3	Rank4	Rank5	Rank6	Rank7	Rank8
NEI	0.000	0.000	0.004	0.048	0.201	0.394	0.263	0.090
AE	0.000	0.001	0.003	0.020	0.066	0.164	0.299	0.448
BDJ	0.015	0.065	0.175	0.333	0.295	0.094	0.021	0.001
WQX	0.340	0.353	0.212	0.075	0.018	0.002	0.000	0.000
YJJ	0.001	0.005	0.015	0.051	0.111	0.186	0.289	0.342
LZJ	0.032	0.110	0.131	0.179	0.193	0.130	0.112	0.113
DW	0.471	0.187	0.155	0.099	0.048	0.019	0.014	0.006
TJQ	0.140	0.279	0.305	0.195	0.068	0.011	0.002	0.000

Notes: TJQ, Taijiquan; BDJ, Baduanjin; WQX, Wuqinxi; YJJ, Yijinjing; LZJ, Liuzijue; DW, Dawu; NEI, non-exercise intervention; AE, aerobic exercise.

**Table A2:** TAG.

	Rank1	Rank2	Rank3	Rank4	Rank5	Rank6	Rank7	Rank8
NEI	0.000	0.000	0.000	0.000	0.004	0.087	0.252	0.657
AE	0.113	0.193	0.214	0.194	0.144	0.095	0.044	0.003
BDJ	0.410	0.290	0.156	0.092	0.037	0.014	0.002	0.000
WQX	0.210	0.229	0.229	0.160	0.105	0.049	0.017	0.001
YJJ	0.108	0.121	0.157	0.196	0.195	0.129	0.079	0.015
LZJ	0.044	0.049	0.068	0.087	0.144	0.233	0.239	0.135
DW	0.078	0.056	0.060	0.080	0.117	0.227	0.227	0.187
TJQ	0.037	0.063	0.116	0.190	0.254	0.140	0.140	0.002

Notes: TJQ, Taijiquan; BDJ, Baduanjin; WQX, Wuqinxi; YJJ, Yijinjing; LZJ, Liuzijue; DW, Dawu; NEI, non-exercise intervention; AE, aerobic exercise.

**Table A3:** HDL-C.

	Rank1	Rank2	Rank3	Rank4	Rank5	Rank6	Rank7	Rank8
NEI	0	0	0	0.001	0.003	0.022	0.144	0.831
AE	0.004	0.013	0.013	0.08	0.143	0.281	0.388	0.05
BDJ	0.147	0.207	0.224	0.202	0.148	0.067	0.006	0
WQX	0.126	0.182	0.182	0.183	0.16	0.095	0.041	0.001
YJJ	0.064	0.08	0.08	0.139	0.168	0.216	0.185	0.043
LZJ	0.382	0.176	0.176	0.096	0.092	0.077	0.05	0.043
DW	0.154	0.161	0.161	0.115	0.12	0.138	0.144	0.063
TJQ	0.123	0.181	0.181	0.184	0.166	0.103	0.042	0

Notes: TJQ, Taijiquan; BDJ, Baduanjin; WQX, Wuqinxi; YJJ, Yijinjing; LZJ, Liuzijue; DW, Dawu; NEI, non-exercise intervention; AE, aerobic exercise.

**Table A4:** LDL-C.

	Rank1	Rank2	Rank3	Rank4	Rank5	Rank6	Rank7	Rank8
NEI	0	0	0	0	0.002	0.032	0.554	0.411
AE	0	0	0.001	0.002	0.008	0.046	0.373	0.57
BDJ	0.049	0.105	0.169	0.232	0.251	0.191	0.003	0
WQX	0.097	0.176	0.227	0.217	0.179	0.101	0.004	0
YJJ	0.159	0.186	0.202	0.165	0.14	0.134	0.012	0.002
LZJ	0.146	0.246	0.155	0.132	0.134	0.152	0.025	0.01
DW	0.497	0.181	0.097	0.073	0.069	0.066	0.012	0.006
TJQ	0.053	0.106	0.149	0.179	0.217	0.279	0.017	0.001

Notes: TJQ, Taijiquan; BDJ, Baduanjin; WQX, Wuqinxi; YJJ, Yijinjing; LZJ, Liuzijue; DW, Dawu; NEI, non-exercise intervention; AE, aerobic exercise.
